# Stent-less percutaneous coronary intervention for a severely calcified coronary lesion using combined orbital atherectomy and intravascular lithotripsy followed by drug-coated balloon: case report

**DOI:** 10.3389/fcvm.2026.1925427

**Published:** 2026-10-08

**Authors:** Toru Misawa, Tetsumin Lee, Takashi Ashikaga, Toshihiro Nozato, Taishi Yonetsu, Tetsuo Sasano

**Affiliations:** 1 Department of Cardiology, Japanese Red Cross Musashino Hospital, Tokyo, Japan; 2 Department of Cardiovascular Medicine, Institute of Science Tokyo, Tokyo, Japan

**Keywords:** coronary calcification, drug-coated balloon, intravascular lithotripsy, orbital atherectomy, percutaneous coronary intervention

## Abstract

**Background:**

Percutaneous coronary intervention (PCI) for severely calcified coronary lesions remains technically challenging. Although the combination of atherectomy and intravascular lithotripsy (IVL) has demonstrated favorable outcomes before drug-eluting stent (DES) implantation, it remains unclear whether a stepwise calcium modification strategy using atherectomy followed by IVL can achieve sufficient lesion preparation to enable a stent-less strategy using drug-coated balloon (DCB) angioplasty.

**Case summary:**

An 83-year-old woman presented with exertional angina despite optimal medical therapy. Coronary angiography revealed severe calcified stenosis in the proximal left anterior descending artery. Pre-procedural optical coherence tomography (OCT) demonstrated thick circumferential calcification, indicating a high risk of inadequate balloon expansion. Orbital atherectomy (OA) was initially performed and achieved partial superficial calcium modification; however, extensive residual calcification remained (post-OA OCT calcium score of 4). Consequently, additional lesion preparation using IVL was performed, delivering 80 pulses via a 2.5-mm IVL balloon, which successfully created multiple calcium fractures and improved vessel compliance. Post-IVL angiography demonstrated residual stenosis <30% with thrombolysis in myocardial infarction grade 3 flow, and fractional flow reserve was 0.92, supporting the decision to avoid DES implantation. Two paclitaxel-coated balloons were subsequently inflated, resulting in an excellent angiographic and OCT outcome without procedural complications. Follow-up coronary angiography and OCT at 17 months demonstrated sustained vessel patency without restenosis, and the patient remained asymptomatic.

**Conclusion:**

This case demonstrates the feasibility of a stepwise, OCT-guided stent-less PCI strategy using sequential OA, IVL, and DCB angioplasty for a severely calcified coronary lesion. The complementary calcium-modification mechanisms of OA and IVL achieved effective lesion preparation, enabling safe deferral of stent implantation and successful DCB angioplasty. Favorable angiographic, OCT, and clinical outcomes at 17 months suggest that this stepwise approach may represent a promising treatment strategy for carefully selected patients with severely calcified coronary lesions and warrants further evaluation in larger clinical studies.

## Introduction

Percutaneous coronary intervention (PCI) for severely calcified coronary lesions remains a major challenge for interventional cardiologists (1, 2). The efficacy and the safety intravascular lithotripsy (IVL) prior to drug-eluting stent (DES) implantation have been well established (3). Moreover, recent studies have demonstrated the feasibility of combining atherectomy devices and IVL before DES implantation in severely calcified lesions (4). However, it remains unclear whether stepwise calcium modification using an atherectomy device followed by IVL can achieve sufficient lesion preparation to enable a stent-less strategy using drug-coated balloon (DCB) angioplasty. Here, we report a case in which serial OCT assessment guided escalation from orbital atherectomy (OA) to IVL because substantial calcification remained after OA, followed by DCB angioplasty after adequate lesion preparation was confirmed by angiography, OCT, and fractional flow reserve (FFR).

## Case presentation

An 83-year-old woman presented to our hospital with exertional chest pain and shortness of breath despite optimal medical therapy for two months. Her symptoms corresponded to Canadian Cardiovascular Society class II angina. She had a 20-year history of hypertension and dyslipidemia that were well controlled. She was a non-smoker and occasionally consumed alcohol. A 12-lead electrocardiogram showed no acute ischemic changes. Echocardiography revealed normal left ventricular functions without regional wall motion abnormalities. Stress myocardial perfusion imaging demonstrated inducible ischemia in the anterior wall of the left ventricle.

Coronary angiography (CAG) revealed a severe stenosis with marked calcification in the proximal left anterior descending artery (LAD) (Figures 1A,B). Unfractionated heparin (100 U/kg) was administered intravenously to maintain an activated clotting time of >250 s throughout the procedure. Pre-PCI OCT demonstrated thick circumferential calcification (360° calcium angle, 1,350 µm calcium thickness, and a 25 mm of calcified segment length), suggesting a high risk of suboptimal balloon expansion (Figures 1C–E). Given the extremely thick calcified plaque with the guidewire adjacent to the calcified segment, OA (Cardiovascular System, Inc, St. Paul, MN, USA) was initially performed for lesion preparation. OA with a 1.25-mm crown was performed 6 times with a cumulative ablation time of 120 s at low rotational speed. Although OCT imaging after OA demonstrated partial calcified plaque ablation, extensive residual calcification remained. The post-OA OCT-based calcium score was 4 (360° calcium angle, 1,110 µm calcium thickness, and a 25 mm calcified segment length), indicating a high risk of inadequate lesion expansion (Figures 2A–D) (5). Therefore, additional lesion modification using IVL was performed. The proximal reference lumen diameter measured by OCT was 2.6 mm, while the proximal reference vessel diameter measured by quantitative coronary angiography was 2.7 mm. The lesion length was 46 mm. A 2.5-mm IVL balloon (Shockwave C2; Shockwave Medical Inc., Santa Clara, CA, USA), corresponding to a balloon-to-artery ratio of 0.93, was used to modify the residual calcification. The IVL balloon was inflated 8 times, and a total of 80 pulses were delivered using a single IVL catheter. Post-IVL angiography and OCT showed acceptable results, including thrombolysis in myocardial infarction (TIMI) grade 3 coronary flow, residual stenosis <30%, and multiple calcium fractures without major coronary dissection (Figures 2E–H). FFR was additionally measured to objectively assess the functional significance of the residual lesion and to support the decision to avoid DES implantation. The post-IVL FFR was 0.92, indicating the absence of functionally significant residual stenosis (Supplementary Figure S1). Subsequently, two paclitaxel-coated balloons (SeQuent Please NEO; Nipro Corporation, Osaka, Japan), 2.5 mm × 25 mm and 3.0 mm × 25 mm, were inflated for 60 s each. The final CAG and OCT findings revealed minimal residual stenosis with TIMI grade 3 coronary flow (Figure 3). The patient remained asymptomatic without periprocedural complications. The patient was prescribed dual antiplatelet therapy consisting of aspirin (100 mg/day) and prasugrel (3.75 mg/day) for 3 months, followed by prasugrel monotherapy (3.75 mg/day). It should be noted that the 3.75 mg/day dose of prasugrel is the standard approved maintenance dose in Japan, based on clinical data suggesting a lower risk of bleeding in East Asian populations compared with Western populations, where a 10 mg/day dose is typically used (6). Follow-up CAG and OCT at 17 months demonstrated no restenosis (Figure 4).

**Figure 1:**
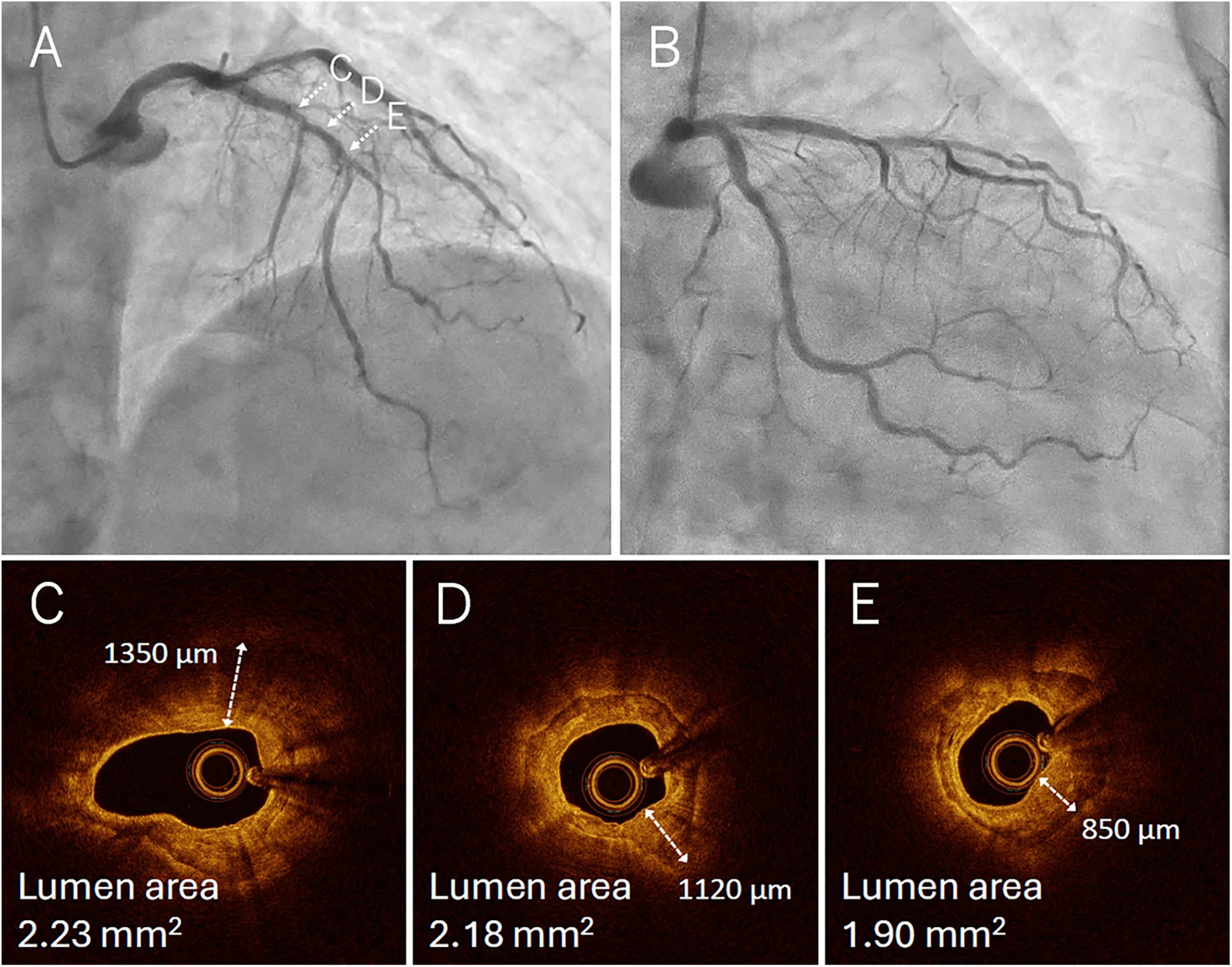
**(A,B)** Pre-procedural coronary angiography shows severe stenosis with heavy calcification in the proximal segment of the left anterior descending artery. **(C–E)** Pre-procedural optical coherence tomography images demonstrate severe circumferential calcification, with calcium thicknesses of 1,350, 1,120, and 850 µm at each cross-section, respectively (white dotted double-headed arrows).

**Figure 2:**
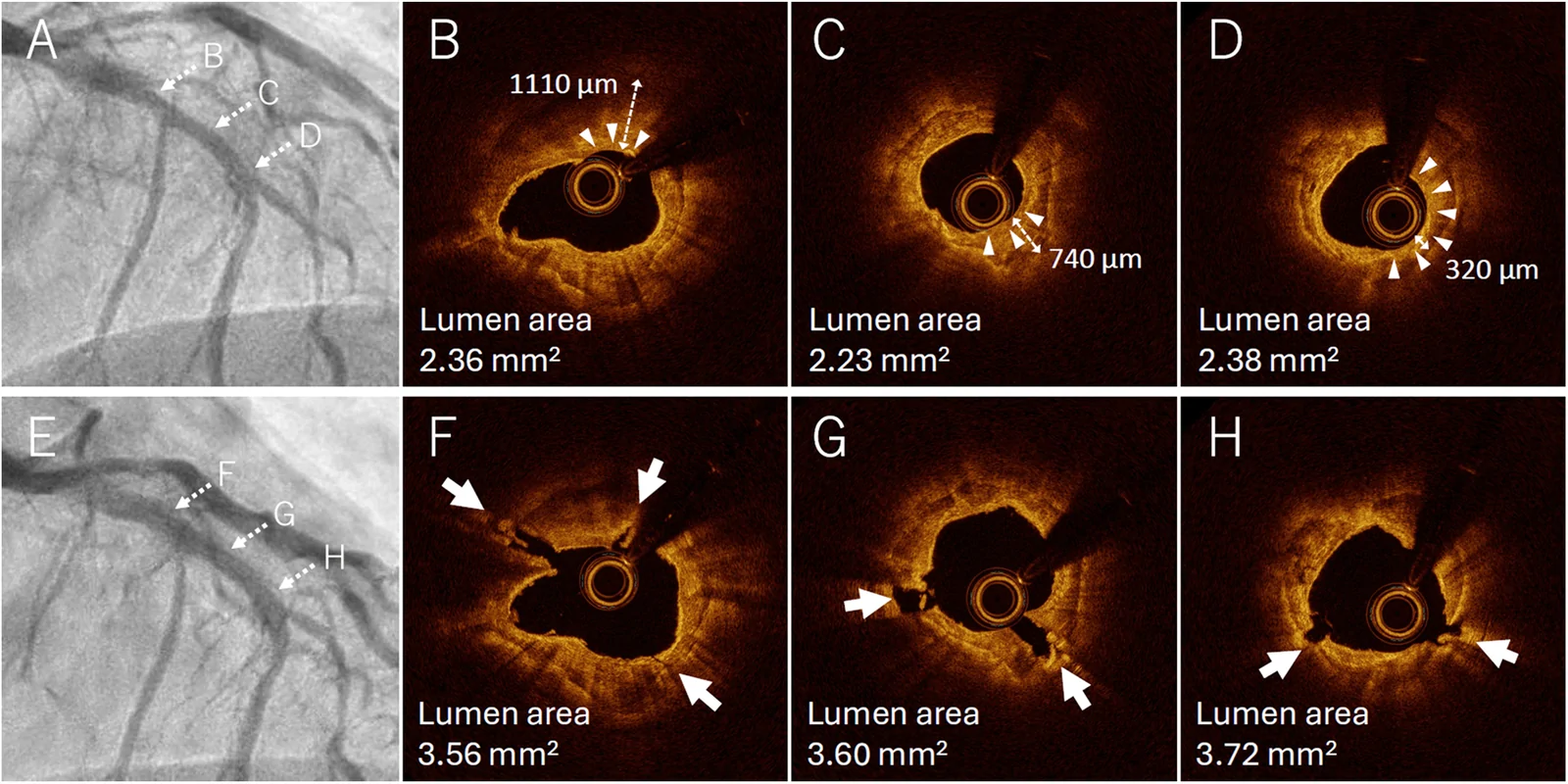
**(A)** Coronary angiography and **(B–D)** optical coherence tomography (OCT) images obtained after orbital atherectomy (OA). The OCT images show debulked areas at the severely calcified lesion following OA (white arrowheads) with calcium thicknesses of 1,110, 740, and 320 µm at each cross-section, respectively (white dotted double-headed arrows). **(E)** Coronary angiography and **(F,G)** OCT images after intravascular lithotripsy. The OCT images show adequate luminal expansion with multiple calcium fractures (white arrows). Panels **B–D** and **F–H** represent matched cross-sectional levels corresponding to panels **C–E** in Figure 1, allowing direct comparison of serial changes in lumen area and calcium morphology.

**Figure 3:**
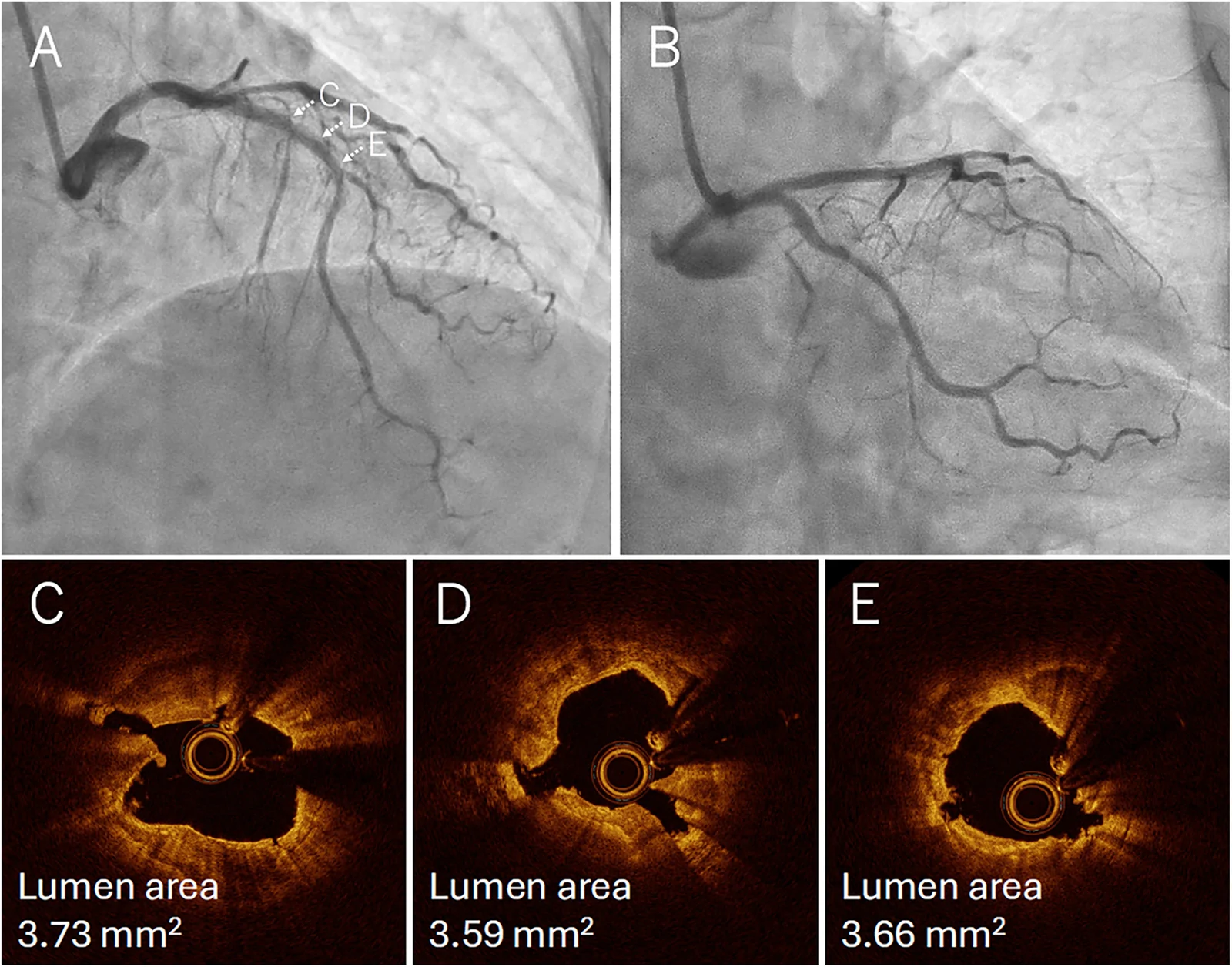
**(A,B)** Coronary angiography and **(C–E)** optical coherence tomography images after percutaneous coronary intervention show no significant residual stenosis and acceptable lumen area without major dissection.

**Figure 4:**
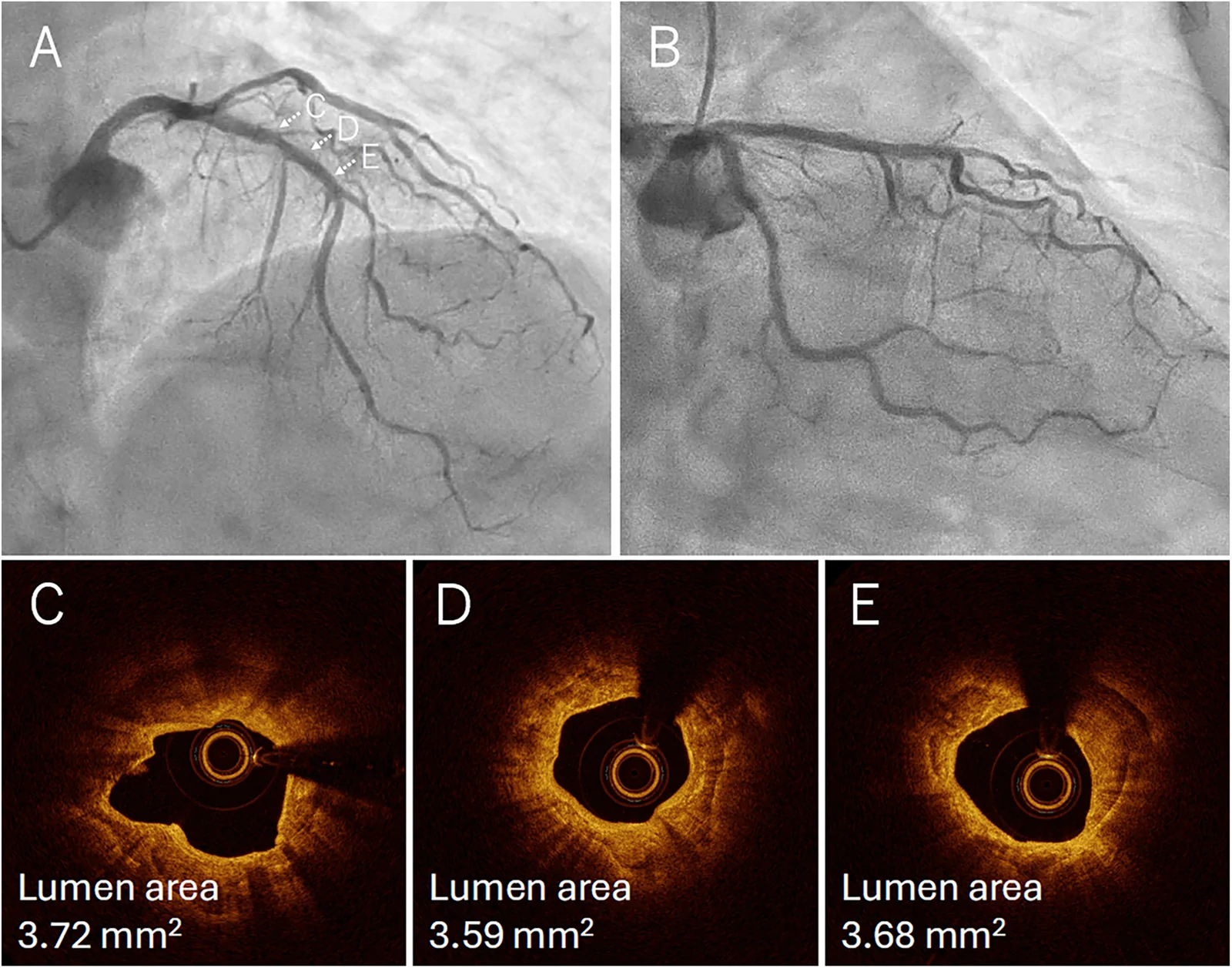
**(A,B)** Follow-up coronary angiography and **(C–E)** optical coherence tomography images at 17 months after the procedure. Restenosis is not observed at the proximal segment of left anterior descending artery.

## Patient perspective

She experienced no periprocedural complications and remained free from exertional chest pain and shortness of breath during the 17-month follow-up period. She expressed satisfaction with the clinical outcome and provided written informed consent for publication of this case report.

## Discussion

We report a case of severely calcified coronary stenosis successfully treated with a combination of OA and IVL followed by DCB angioplasty. The principal clinical insight of this case is that serial OCT can guide a stepwise lesion preparation strategy by demonstrating the complementary effect of OA and IVL, thereby facilitating the selection of a stent-less PCI approach after adequate lesion preparation. To the best of our knowledge, this is the first report describing a stent-less PCI strategy using the three-step combination of OA, IVL, and DCB angioplasty, with serial OCT assessment demonstrating the morphological effects of each treatment stage and long-term angiographic and OCT follow-up.

Severely calcified coronary lesions are associated with adverse clinical outcomes and remain a significant challenge during PCI. Adequate lesion preparation is essential to achieve sufficient luminal expansion and optimal procedural outcomes, particularly when a stent-less strategy using DCB is considered. Atherectomy devices can facilitate effective plaque modification in severely calcified lesions and may improve procedural outcomes in this setting. Previous studies have demonstrated favorable angiographic and clinical outcomes with a stent-less strategy using atherectomy followed by DCB angioplasty in patients with *de novo* coronary lesions (7, 8).

However, achieving adequate plaque modification in severely calcified lesions is particularly challenging when thick or deep calcium is present. IVL has recently emerged as a novel modality capable of fracturing deep calcium through the generation of acoustic pressure waves. In this context, the combination of atherectomy devices and IVL may provide complementary mechanisms of calcium modification. Atherectomy primarily modifies superficial calcium and facilitates device delivery, whereas IVL can effectively fracture deeper calcium sheets. Although rotational atherectomy (RA) was also considered a reasonable option, OA was selected because the guidewire position provided favorable guidewire bias. The guidewire was located adjacent to the calcified plaque, allowing effective modification of the superficial calcified component. In addition, OA was expected to serve as the first step of a stepwise lesion preparation strategy to facilitate subsequent IVL balloon deliverability should substantial residual calcification remain after atherectomy. Nevertheless, this case does not establish the superiority of OA over RA. Rather, the choice of atherectomy device should be individualized according to lesion morphology, guidewire position, and the anticipated procedural strategy.

Previous studies have demonstrated the feasibility of combining atherectomy and IVL prior to DES implantation in severely calcified lesions (4). Furthermore, several studies have suggested that a stent-less strategy using DCB after IVL may also be feasible in selected patients with severely calcified *de novo* coronary lesions, although DCB use after IVL remains off-label (9–11). A previous study reported that target lesion failure, defined as a composite of culprit lesion-related cardiac death, non-fatal myocardial infarction and target lesion revascularization, occurred in 8.9% of patients treated with IVL followed by DCB angioplasty (10). Another study demonstrated that DCB-only angioplasty without stent implantation was possible in 75% of calcified lesions, with only one case of binary restenosis at the 4-month angiographic follow-up among 35 patients (11). Nevertheless, these studies did not incorporate atherectomy before IVL.

In the present case, serial OCT demonstrated the distinct contribution of each calcium-modification modality. OA primarily modified superficial calcium and facilitated device delivery, whereas IVL produced additional calcium fractures that further improved vessel compliance. Following this stepwise approach to lesion preparation, adequate luminal expansion was achieved, and both OCT and FFR confirmed satisfactory anatomical and physiological results, allowing safe deferral of stent implantation in favor of DCB angioplasty. In accordance with current consensus recommendations, DCB angioplasty was considered appropriate because optimal lesion preparation was achieved, with sufficient lesion expansion and no significant flow-limiting complications (12). No angiographic or OCT evidence of restenosis was observed during the 17-month follow-up. These findings suggest that the complementary effects of OA and IVL may optimize lesion preparation sufficiently to enable an effective stent-less PCI strategy in carefully selected patients.

This case report has several limitations. First, this was a single-case experience, and the feasibility and clinical effectiveness of a stent-less strategy using combined OA and IVL followed by DCB angioplasty cannot be generalized. Second, although follow-up CAG and OCT demonstrated the absence of restenosis at 17 months, follow-up functional assessment was not performed. Third, the use of DCB after combined calcium-modification therapy remains off-label, and the optimal patient selection, procedural criteria, and antiplatelet regimen have not been established. Fourth, OCT images were analyzed by investigators and were not evaluated by an independent core laboratory or blinded readers. Therefore, the quantitative OCT findings should be interpreted cautiously.

Although calcium modification by IVL-generated acoustic pressure waves may induce morphological changes and potentially enhance drug permeability, the precise mechanisms underlying the feasibility of DCB use after combined atherectomy and IVL are incompletely understood. Despite these limitations, the present case supports the potential feasibility of a stepwise, OCT-guided stent-less PCI strategy after complementary calcium modification with OA and IVL. Further studies with larger patient cohorts are warranted to assess the safety, efficacy, and long-term clinical outcomes of this stepwise stent-less PCI strategy.

## Conclusions

A stepwise lesion preparation strategy using OA followed by IVL achieved effective calcium modification in a severely calcified coronary lesion. Serial OCT demonstrated the complementary morphological effects of each modality, enabling optimization of lesion preparation before DCB angioplasty. Based on the favorable anatomical and physiological results after calcium modification, stent implantation was safely deferred, and DCB angioplasty was successfully performed. Follow-up CAG and OCT at 17 months demonstrated sustained vessel patency without restenosis and favorable clinical outcomes. These findings suggest that a stepwise, OCT-guided approach combining atherectomy and IVL may optimize lesion preparation and facilitate a stent-less PCI strategy in carefully selected patients with severely calcified coronary lesions.

## Data Availability

The datasets presented in this article are not readily available because the data are not publicly available due to patient privacy and ethical restrictions. Requests to access the datasets should be directed to Toru Misawa, misawa.toru112@gmail.com.

## Supplementary material

SUpplementary Figure 1 Fractional flow reserve (FFR) assessment after lesion preparationFFR after stepwise lesion preparation was 0.92, indicating the absence of functionally significant residual stenosis.
